# Supplementary material for: Risk of aortic aneurysm and dissection following exposure to fluoroquinolones, common antibiotics, and febrile illness using a self-controlled case series study design: Retrospective analyses of three large healthcare databases in the US
Source: PLoS One. 2021 Aug 16;16(8):e0255887. doi: 10.1371/journal.pone.0255887 (PMC8366987; doi:10.1371/journal.pone.0255887)
Supplement: S1 Table — (RTF) [file pone.0255887.s001.rtf]

S1 Table: Exposure timeline analysis: IRR Estimates for AAD in OPTUMEXTDOD; Risk Window = Exposures Period + 30 days
Exposure	IRR (60d to 30d)	95% CI LB (60d to 30d)	95% CI UB (60d to 30d)	IRR (29d to 1d)	95% CI LB (29d to 1d)	95% CI UB (29d to 1d)	IRR	95% CI LB	95% CI UB	p	Calibrated p	
FQ class	1.396	1.286	1.512	2.671	2.507	2.842	1.355	1.266	1.451	0.000	0.857	
FINTA	1.147	0.614	1.948	1.784	1.095	2.749	4.400	3.216	5.902	0.000	0.000	
Amoxicillin	1.186	1.075	1.306	1.306	1.189	1.432	1.029	0.943	1.120	0.521	0.291	
Azithromycin	0.893	0.780	1.018	0.836	0.728	0.956	1.136	1.023	1.259	0.016	0.511	
Trimethoprim without Sulfamethoxazole	1.014	0.481	1.861	0.221	0.037	0.690	0.683	0.384	1.125	0.164	0.068	
Trimethoprim with Sulfamethoxazole	1.877	1.597	2.191	2.651	2.310	3.030	1.019	0.864	1.195	0.819	0.292	
Key: IRR = Incidence rate ratio, CI = Confidence Interval, LB = Lower Bound, UB = Upper Bound, FINTA = Febrile illness untreated with antibiotics, 
p = p-value, Calibrated p = Empirically Calibrated p-value	
