# Supplementary material for: Risk of aortic aneurysm and dissection following exposure to fluoroquinolones, common antibiotics, and febrile illness using a self-controlled case series study design: Retrospective analyses of three large healthcare databases in the US
Source: PLoS One. 2021 Aug 16;16(8):e0255887. doi: 10.1371/journal.pone.0255887 (PMC8366987; doi:10.1371/journal.pone.0255887)
Supplement: S2 Table — (RTF) [file pone.0255887.s002.rtf]

S2 Table: Exposure timeline analysis: IRR Estimates for AAD in IBMMDCR; Risk Window = Exposures Period + 30 days
Exposure	IRR (60d to 30d)	95% CI LB (60d to 30d)	95% CI UB (60d to 30d)	IRR (29d to 1d)	95% CI LB (29d to 1d)	95% CI UB (29d to 1d)	IRR	95% CI LB	95% CI UB	p	Calibrated p	
FQ class	1.283	1.172	1.401	2.378	2.216	2.549	1.204	1.115	1.298	0.000	0.816	
FINTA	1.211	0.369	2.921	1.154	0.351	2.784	1.553	0.600	3.296	0.311	0.579	
Amoxicillin	1.069	0.958	1.190	1.200	1.080	1.329	0.933	0.846	1.028	0.165	0.067	
Azithromycin	0.765	0.653	0.890	0.707	0.600	0.826	0.958	0.847	1.080	0.492	0.114	
Trimethoprim without Sulfamethoxazole	1.683	0.934	2.782	0.823	0.350	1.617	0.328	0.138	0.654	0.005	0.002	
Trimethoprim with Sulfamethoxazole	1.776	1.501	2.086	2.378	2.050	2.744	1.151	0.978	1.345	0.085	0.753	
Key: IRR = Incidence rate ratio, CI = Confidence Interval, LB = Lower Bound, UB = Upper Bound, FINTA = Febrile illness untreated with antibiotics, p = p-value, Calibrated p = Empirically Calibrated p-value	
	
