# Supplementary material for: Risk of aortic aneurysm and dissection following exposure to fluoroquinolones, common antibiotics, and febrile illness using a self-controlled case series study design: Retrospective analyses of three large healthcare databases in the US
Source: PLoS One. 2021 Aug 16;16(8):e0255887. doi: 10.1371/journal.pone.0255887 (PMC8366987; doi:10.1371/journal.pone.0255887)
Supplement: S3 Table — (RTF) [file pone.0255887.s003.rtf]

S3 Table: Exposure timeline analysis: IRR Estimates for AAD in IBMCOM; Risk Window = Exposures Period + 30 days
Exposure	IRR (60d to 30d)	95% CI LB (60d to 30d)	95% CI UB (60d to 30d)	IRR (29d to 1d)	95% CI LB (29d to 1d)	95% CI UB (29d to 1d)	IRR	95% CI LB	95% CI UB	p	Calibrated p	
FQ class	1.483	1.266	1.726	3.452	3.085	3.852	1.846	1.635	2.078	0.000	0.243	
FINTA	0.330	0.054	1.069	0.333	0.054	1.077	0.642	0.195	1.550	0.403	0.271	
Amoxicillin	1.090	0.973	1.216	1.075	0.959	1.202	1.175	1.068	1.289	0.001	0.799	
Azithromycin	0.759	0.628	0.908	0.665	0.542	0.805	1.274	1.115	1.451	0.000	0.871	
Trimethoprim without Sulfamethoxazole	0.275	0.016	1.236	1.124	0.342	2.701	0.622	0.189	1.500	0.368	0.249	
Trimethoprim with Sulfamethoxazole	1.812	1.468	2.213	3.392	2.884	3.967	1.239	1.016	1.496	0.030	0.863	
Key: IRR = Incidence rate ratio, CI = Confidence Interval, LB = Lower Bound, UB = Upper Bound, FINTA = Febrile illness untreated with antibiotics, p = p-value, Calibrated p = Empirically Calibrated p-value	
