# Supplementary material for: Risk of aortic aneurysm and dissection following exposure to fluoroquinolones, common antibiotics, and febrile illness using a self-controlled case series study design: Retrospective analyses of three large healthcare databases in the US
Source: PLoS One. 2021 Aug 16;16(8):e0255887. doi: 10.1371/journal.pone.0255887 (PMC8366987; doi:10.1371/journal.pone.0255887)
Supplement: S7 Table — Risk Window = Exposure period + 60 Days, Database = OPTUMEXTDOD. (RTF) [file pone.0255887.s007.rtf]

S7 Table: Sensitivity analysis: IRR Estimate for AAD in a subset of the primary population that did not have an inpatient hospitalization with a discharge date within 30 days of AAD. Risk Window = Exposure period + 60 Days, Database = OPTUMEXTDOD
Exposure	IRR	95% CI LB	95% CI UB	P	Calibrated p	
FQ class	0.975	0.910	1.042	0.457	0.194	
FINTA	2.234	1.573	3.09	0.000	0.009	
Amoxicillin	0.924	0.855	0.998	0.046	0.109	
Azithromycin	0.997	0.906	1.095	0.948	0.258	
Trimethoprim without Sulfamethoxazole	0.823	0.498	1.284	0.420	0.191	
Trimethoprim with Sulfamethoxazole	0.749	0.637	0.874	0.000	0.008	
Key: IRR = Incidence rate ratio, CI = Confidence Interval, LB = Lower Bound, UB = Upper Bound, FINTA = Febrile illness untreated with antibiotics, p = p-value, Calibrated p = Empirically Calibrated p-value	
	
