# Supplementary material for: Risk of aortic aneurysm and dissection following exposure to fluoroquinolones, common antibiotics, and febrile illness using a self-controlled case series study design: Retrospective analyses of three large healthcare databases in the US
Source: PLoS One. 2021 Aug 16;16(8):e0255887. doi: 10.1371/journal.pone.0255887 (PMC8366987; doi:10.1371/journal.pone.0255887)
Supplement: S10 Table — Risk Window = Exposure period + 30 Days, Database = OPTUMEXTDOD. (RTF) [file pone.0255887.s010.rtf]

S10 Table: Sensitivity analysis: IRR Estimate for AAD in a subset of the primary population that did not have an inpatient hospitalization with a discharge date within 60 days of AAD. Risk Window = Exposure period + 30 Days, Database = OPTUMEXTDOD
Exposure	IRR	95% CI LB	95% CI UB	P	Calibrated p	
FQ class	1.426	1.328	1.530	0.000	0.693	
FINTA	4.506	3.310	6.020	0.000	0.000	
Amoxicillin	1.113	0.988	1.250	0.075	0.498	
Azithromycin	1.242	1.123	1.372	0.000	0.809	
Trimethoprim without Sulfamethoxazole	0.464	0.123	1.317	0.205	0.111	
Trimethoprim with Sulfamethoxazole	1.020	0.870	1.188	0.807	0.309	
Key: IRR = Incidence rate ratio, CI = Confidence Interval, LB = Lower Bound, UB = Upper Bound, FINTA = Febrile illness untreated with antibiotics, p = p-value, Calibrated p = Empirically Calibrated p-value	
