# Supplementary material for: Risk of aortic aneurysm and dissection following exposure to fluoroquinolones, common antibiotics, and febrile illness using a self-controlled case series study design: Retrospective analyses of three large healthcare databases in the US
Source: PLoS One. 2021 Aug 16;16(8):e0255887. doi: 10.1371/journal.pone.0255887 (PMC8366987; doi:10.1371/journal.pone.0255887)
Supplement: S11 Table — Risk Window = Exposure period + 30 Days, Database = IBMMDCR. (RTF) [file pone.0255887.s011.rtf]

S11 Table: Sensitivity analysis: IRR Estimate for AAD in a subset of the primary population that did not have an inpatient hospitalization with a discharge date within 60 days of AAD. Risk Window = Exposure period + 30 Days, Database = IBMMDCR
Exposure	IRR	95% CI LB	95% CI UB	P	Calibrated p	
FQ class	1.279	1.180	1.385	0.000	0.364	
FINTA	1.490	0.577	3.152	0.358	0.606	
Amoxicillin	1.095	0.950	1.256	0.203	0.440	
Azithromycin	1.039	0.920	1.169	0.530	0.165	
Trimethoprim without Sulfamethoxazole	0.107	0.006	0.513	0.049	0.035	
Trimethoprim with Sulfamethoxazole	1.156	0.987	1.345	0.067	0.772	
Key: IRR = Incidence rate ratio, CI = Confidence Interval, LB = Lower Bound, UB = Upper Bound, FINTA = Febrile illness untreated with antibiotics, p = p-value, Calibrated p = Empirically Calibrated p-value	
