# Supplementary material for: Risk of aortic aneurysm and dissection following exposure to fluoroquinolones, common antibiotics, and febrile illness using a self-controlled case series study design: Retrospective analyses of three large healthcare databases in the US
Source: PLoS One. 2021 Aug 16;16(8):e0255887. doi: 10.1371/journal.pone.0255887 (PMC8366987; doi:10.1371/journal.pone.0255887)
Supplement: S12 Table — Risk Window = Exposure period + 30 Days, Database = IBMCOM. (RTF) [file pone.0255887.s012.rtf]

S12 Table: Sensitivity analysis: IRR Estimate for AAD in a subset of the primary population that did not have an inpatient hospitalization with a discharge date within 60 days of AAD. Risk Window = Exposure period + 30 Days, Database = IBMCOM
Exposure	IRR	95% CI LB	95% CI UB	P	Calibrated p	
FQ class	1.684	1.510	1.872	0.000	0.269	
FINTA	0.708	0.216	1.691	0.510	0.380	
Amoxicillin	1.012	0.862	1.181	0.879	0.575	
Azithromycin	1.321	1.155	1.506	0.000	0.755	
Trimethoprim without Sulfamethoxazole	0.000	-	-	-	-	
Trimethoprim with Sulfamethoxazole	1.022	0.835	1.240	0.828	0.604	
Key: IRR = Incidence rate ratio, CI = Confidence Interval, LB = Lower Bound, UB = Upper Bound, FINTA = Febrile illness untreated with antibiotics, p = p-value, Calibrated p = Empirically Calibrated p-value	
	
