# Supplementary material for: Risk of aortic aneurysm and dissection following exposure to fluoroquinolones, common antibiotics, and febrile illness using a self-controlled case series study design: Retrospective analyses of three large healthcare databases in the US
Source: PLoS One. 2021 Aug 16;16(8):e0255887. doi: 10.1371/journal.pone.0255887 (PMC8366987; doi:10.1371/journal.pone.0255887)
Supplement: S13 Table — Risk Window = Exposure period + 30 Days, Database = OPTUMEXTDOD. (RTF) [file pone.0255887.s013.rtf]

S13 Table: Sensitivity analysis: IRR Estimate for AAD, controlling for other concurrent drugs. Risk Window = Exposure period + 30 Days, Database = OPTUMEXTDOD
Exposure	IRR	95% CI LB	95% CI UB	p	Calibrated p	
FQ class	1.241	1.152	1.336	0.000	0.839	
FINTA	4.422	3.234	5.944	0.000	0.000	
Amoxicillin	0.998	0.842	1.231	0.980	0.441	
Azithromycin	1.092	0.984	1.208	0.094	0.669	
Trimethoprim without Sulfamethoxazole	0.430	0.113	1.233	0.166	0.114	
Trimethoprim with Sulfamethoxazole	0.868	0.739	1.014	0.081	0.168	
Key: IRR = Incidence rate ratio, CI = Confidence Interval, LB = Lower Bound, UB = Upper Bound, FINTA = Febrile illness untreated with antibiotics, p = p-value, Calibrated p = Empirically Calibrated p-value	
