# Supplementary material for: Risk of aortic aneurysm and dissection following exposure to fluoroquinolones, common antibiotics, and febrile illness using a self-controlled case series study design: Retrospective analyses of three large healthcare databases in the US
Source: PLoS One. 2021 Aug 16;16(8):e0255887. doi: 10.1371/journal.pone.0255887 (PMC8366987; doi:10.1371/journal.pone.0255887)
Supplement: S14 Table — Risk Window = Exposure period + 30 Days, Database = IBMMDCR. (RTF) [file pone.0255887.s014.rtf]

S14 Table: Sensitivity analysis: IRR Estimate for AAD, controlling for other concurrent drugs. Risk Window = Exposure period + 30 Days, Database = IBMMDCR
Exposure
	IRR	95% CI LB	95% CI UB	p	Calibrated p	
FQ class	1.034	0.951	1.122	0.432	0.086	
FINTA	1.544	0.582	3.386	0.333	0.513	
Amoxicillin	0.904	0.805	1.006	0.076	0.001	
Azithromycin	0.914	0.803	1.037	0.169	0.004	
Trimethoprim without Sulfamethoxazole	0.344	0.134	0.718	0.013	0.005	
Trimethoprim with Sulfamethoxazole	0.977	0.824	1.151	0.788	0.0993	
Key: IRR = Incidence rate ratio, CI = Confidence Interval, LB = Lower Bound, UB = Upper Bound, FINTA = Febrile illness untreated with antibiotics, p = p-value, Calibrated p = Empirically Calibrated p-value	
