# Supplementary material for: Risk of aortic aneurysm and dissection following exposure to fluoroquinolones, common antibiotics, and febrile illness using a self-controlled case series study design: Retrospective analyses of three large healthcare databases in the US
Source: PLoS One. 2021 Aug 16;16(8):e0255887. doi: 10.1371/journal.pone.0255887 (PMC8366987; doi:10.1371/journal.pone.0255887)
Supplement: S15 Table — Risk Window = Exposure period + 30 Days, Database = IBMCOM. (RTF) [file pone.0255887.s015.rtf]

S15 Table: Sensitivity analysis: IRR Estimate for AAD, controlling for other concurrent drugs. Risk Window = Exposure period + 30 Days, Database = IBMCOM
Exposure	IRR	95% CI LB	95% CI UB	p	Calibrated p	
FQ class	1.382	1.203	1.583	0.000	0.728	
						
Amoxicillin	1.223	1.087	1.371	0.001	0.871	
Azithromycin	1.212	1.049	1.394	0.008	0.877	
Trimethoprim without Sulfamethoxazole	0.701	0.210	1.735	0.510	0.456	
Trimethoprim with Sulfamethoxazole	0.707	0.562	0.880	0.002	0.262	
Febrile illness untreated with antibiotics	0.493	0.118	1.341	0.255	0.255	
Key: IRR = Incidence rate ratio, CI = Confidence Interval, LB = Lower Bound, UB = Upper Bound, FINTA = Febrile illness untreated with antibiotics, p = p-value, Calibrated p = Empirically Calibrated p-value	
