# Supplementary material for: Risk of aortic aneurysm and dissection following exposure to fluoroquinolones, common antibiotics, and febrile illness using a self-controlled case series study design: Retrospective analyses of three large healthcare databases in the US
Source: PLoS One. 2021 Aug 16;16(8):e0255887. doi: 10.1371/journal.pone.0255887 (PMC8366987; doi:10.1371/journal.pone.0255887)
Supplement: Attachment — Submitted filename: AA Reviewer Comments PLOS One RESPONSE FINAL.docx [file pone.0255887.s016.docx]

*Response to Reviewers*

Additional Editor Comments:

A more detailed explanation between calibrated p values and traditional p values is needed for the reader to understand their differences. Besides, it will be better to re-arrange the order of the findings in the result sections as the reviewer's suggestion.

**A paragraph explaining the negative control approach, and references to the p-value calibration work, had been added in the introduction: “****Another key limitation of prior analyses is the possibility of systemic bias. Residual bias can occur in all large retrospective database analyses after confounding control has been implemented and this bias can skew results in even the best designed studies. Approaches to identify residual bias often include the use of negative controls – exposures known to not cause the outcome of interest. The distribution of effects obtained from analyzing a large number of negative controls can be utilized to create a so-called calibrated p-value, one that, based on the data that we are actually using – rather than *a priori* statistical considerations – reflects the probability that the observed effect would be seen by chance. (11-13) For example, if the negative controls are not centered on the null value or are more scattered than expected, the calibrated p value would take this into account. Further details are added in the discussion section.”**

 The authors calculated event occurrence before and after the first FQ exposure and estimated the incidence rate ratio (IRR) before the first FQ exposure to demonstrate there may be potential confounding for the association between FQ and AA/AD (Table 2 and Figure 2).
However, the timeline scales mentioned in the methods (e.g., within “60 days” before and after the first FQ exposure) and shown in the results (e.g., within “150 days” before and after the first FQ exposure in Figure 2) were not consistent, which may make the audience a little bit confusing. It will be better to have consistent timeline scales in the method and result sections.

**Figure 2 was modified to include only the 60-day pre- and post-exposure time period, to be consistent with the at-risk definitions described in the methods.**

2. The authors estimated calibrated p values for FQ, febrile illness not treated with antibiotics, amoxicillin, azithromycin, trimethoprim/sulfamethoxazole, and 38 negative exposure controls to demonstrate there may be bias or systematic errors for the association between FQ and AA/AD.
However, for the general audience, it is not easy or intuitive to understand why the measure of calibrated p values can indicate potential bias. It will be better to have more detailed explanation for the difference between calibrated p values and traditional p values and why calibrated p values can suggest potential bias in the methods section.

**A paragraph explaining the methodology was added in the introduction (See above). In addition, greater discussion of our approach, and the rationale thereof, was added in the Discussion section: “In our study and as shown in Figure 1, the relative risks obtained from using negative controls were not centered around the null, as would be expected in a study with no residual bias. Negative control estimates were instead centered between 1 and 2, suggesting a moderate, positive systemic error. In Figures 1A-1C: the dashed lines represent thresholds of significance using non-calibrated p value: any estimate below the dashed lines, in a study with no residual bias, would be considered significant. In contrast, the estimates in the solid orange area have calibrated p < 0.05. Using non-calibrated p values, 8 out of 38 negative controls would have yielded significant risk estimates for AAD in OPTUMEXTDOD versus 2 out of 38 using calibrated p values. Calibrating p value was therefore essential to control for systemic error in our databases. “**

3. The authors mentioned they estimated IRR for FQ, febrile illness not treated with antibiotics, amoxicillin, azithromycin, and trimethoprim/sulfamethoxazole, followed by calibration of p values for 38 negative exposure controls and so called pre-exposure and timeline analyses. However, the results did not show up as this order, which made the article difficult to read and follow-up. It will be better to re-arrange the order of the findings (including main text, figures, and tables) in the result sections.

**The findings were reorganized accordingly.**

4. It will be better to indicate “Appendix table 1, Appendix table 2, and etc.” in the main text and in the supplemental materials, which will be better for the audience to find corresponding results.

**Appendix Tables were relabeled accordingly.**
